# Extensive ischemic gastroduodenal necrosis caused by antihypertensive drug overdose in a young man

**DOI:** 10.1002/ccr3.8348

**Published:** 2023-12-22

**Authors:** Tao Yuan, Ji Sun, Qin Ma

**Affiliations:** ^1^ Department of Anesthesiology, West China Hospital Sichuan University Chengdu China; ^2^ Institute of Integrated Traditional Chinese and Western Medicine, West China Hospital, Sichuan University Chengdu China; ^3^ Division of Gastrointestinal Surgery, Department of General Surgery, West China Hospital Sichuan University Chengdu China

**Keywords:** acute abdomen, drug overdose, gastroduodenal necrosis, hepatic portal venous gas sign

## Abstract

To raise the awareness of the rare and life‐threatening clinical entity, we report a 23‐year‐old male presenting with acute abdomen who was finally diagnosed with gastroduodenal necrosis due to gut hypoperfusion after antihypertensive drug overdose.

## Graphical abstract

A 23‐year‐old Chinese male presenting with acute abdomen and hepatic portal venous gas sign was intraoperatively diagnosed with extensive ischemic gastroduodenal necrosis due to gut hypoperfusion after antihypertensive drug overdose.

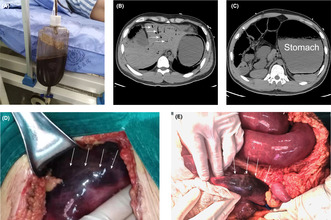

## 1 CASE PRESENTATION

A 23‐year‐old Chinese male presented to the emergency department with persistent diffuse abdominal pain for 2 days after overdose intake of antihypertensive drugs (including 60 nifedipine tablets, 14 olmesartan tablets, and 50 clonidine tablets). He had a medical history of nephrotic syndrome for 18 years and episode of secondary acute left cerebral infarct 2 years ago. Long‐lasting illness and suffering caused him depression and even suicide attempts. Vital signs were within normal limits (conscious; temperature, 36.7°C; heart rate, 74 beats per minute; respiratory rate, 19 beats per minute; blood pressure, 109/66 mmHg). Physical examination result was remarkable for whole abdominal tenderness with rebounding pain.

**FIGURE 1:**
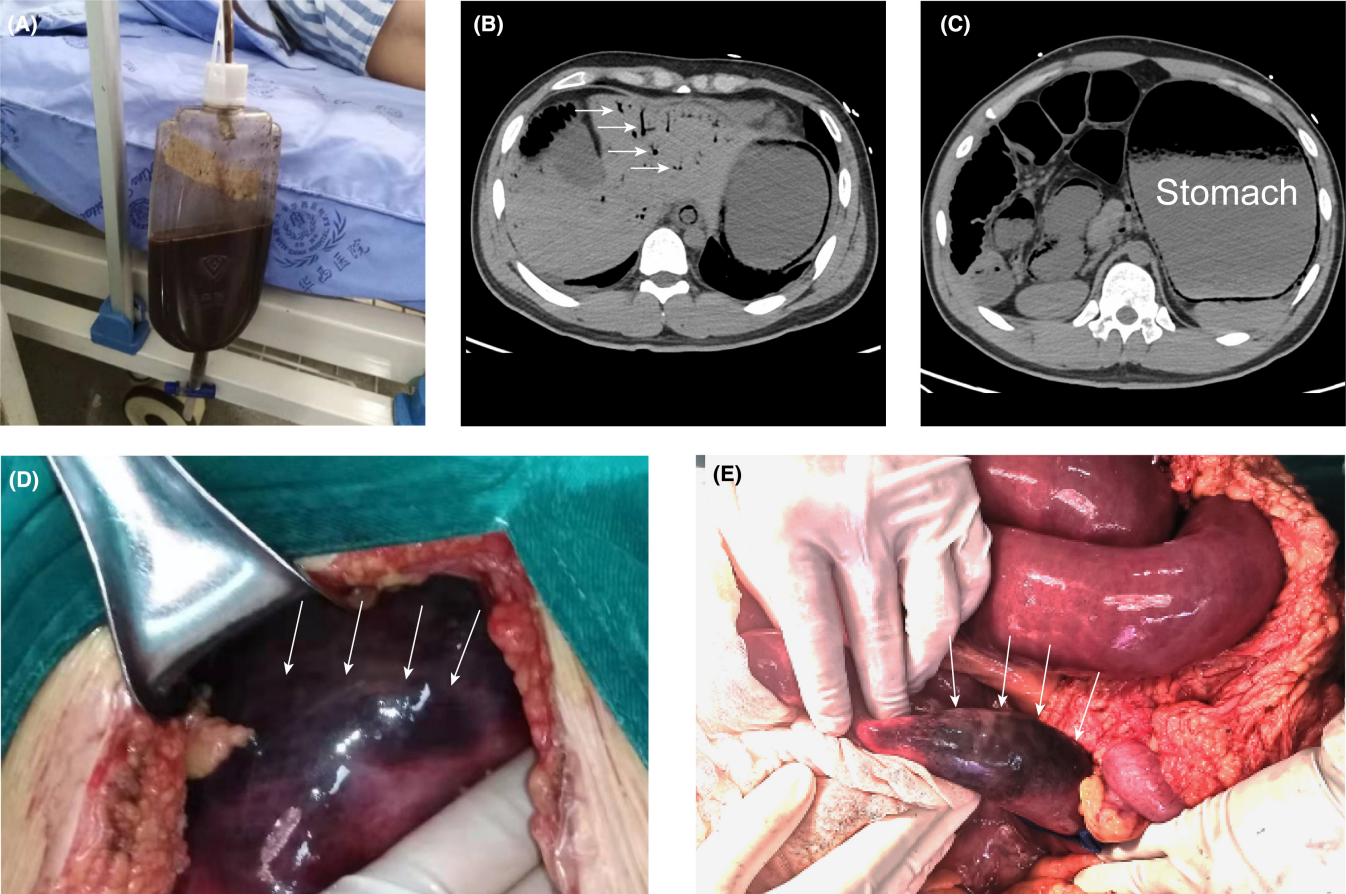
(A) Dark‐red bloody stomach content in the gastrointestinal decompression apparatus; computed tomography of the abdomen demonstrating (B) hepatic portal venous gas sign (arrow) and (C) severe gastrointestinal tract dilatation. (D, E) Intraoperative photographs revealing gastrointestinal tract necrosis from cardia to proximal 15 cm of the jejunum (arrow).

After nasogastric tube placement, dark‐red bloody stomach content was seen as shown in Figure 1A. Laboratory tests revealed a hemoglobin count of 132 g/L, white blood cell count of 6.81 × 10^9^/L (82.8% neutrophils), abnormal liver function, and elevated blood urea nitrogen and lactate (2.4 mmol/L) levels. Computed tomography (CT) of the abdomen (Figure 1B,C) demonstrated hepatic portal venous gas (HPVG) sign and severe gastrointestinal tract (GIT) dilatation, implicating the potential diagnosis of GIT ischemia and necrosis, which was subsequently confirmed by urgent exploratory laparotomy (Figure 1D,E).

## 2 DIAGNOSIS

Gastroduodenal ischemic necrosis.

During surgical exploration, GIT necrosis from cardia to proximal 15 cm of the jejunum, significant stomach and small bowel dilatation, and plenty of palpable tablets in the transversus and descending colon were found. The eroded and pale mucosa of lower esophagus was detected by intraoperative upper endoscopy examination. After much cogitation, the patient's father refused further operative treatment and requested voluntary discharge due to the traditional Chinese conception of dying at home.

## 3 DISCUSSION

HPVG sign is a rare and concerning CT finding and often must be urgently managed, especially by surgery.^1^ Extensive ischemic bowel necrosis has proven to be the most common cause of HPVG (approximately 72%) and predicts increased risk of mortality (>50%).^2^ Gastroduodenal necrosis (GDN), however, is a relatively rare and life‐threatening condition in adults because the stomach and duodenum has a rich innate collateral blood supply.^3^

For this young man, gut hypoperfusion secondary to hypotension due to antihypertensive drug overdose might be the indeed cause of GDN. The present case report aims to raise the physicians' awareness and recognition of gastrointestinal ischemia due to drug overdose when checking acute abdomen patients and to appeal to manufacturers to identify and elucidate severe adverse events of each antihypertensive drug.

## AUTHOR CONTRIBUTIONS

**Tao Yuan:** Writing – original draft. **Ji Sun:** Validation; visualization; writing – review and editing. **Qin Ma:** Supervision; validation; writing – review and editing.

## FUNDING INFORMATION

This research was funded by Sichuan Provincial Administration of Traditional Chinese Medicine (Grant No. 2023MS173).

## CONFLICT OF INTEREST STATEMENT

There is no conflict of interest to disclose.

## ETHICS STATEMENT

The present study involving human subjects was reviewed and approved by Ethical Committee on Biomedical Research, West China Hospital, Sichuan University (No. 2023‐217).

## CONSENT

Written informed consent was obtained from the individual for the publication of any potentially identifiable images or data included in this article.

## Data Availability

Due to privacy and ethical restrictions, the data supporting the findings of this study are available from the corresponding author only upon reasonable request.
